# Effect of enhanced detailing and mass media on community use of oral rehydration salts and zinc during a scale-up program in Gujarat and Uttar Pradesh

**DOI:** 10.7189/jogh.09.010501

**Published:** 2019-06

**Authors:** Felix Lam, George Pro, Shreya Agrawal, Vishal Dev Shastri, Leslie Wentworth, Melinda Stanley, Nitin Beri, Abhishek Tupe, Ashutosh Mishra, Hamsa Subramaniam, Kate Schroder, Marta Rose Prescott, Naresh Trikha

**Affiliations:** 1 Clinton Health Access Initiative, Boston, Massachusetts, USA; 2 Clinton Health Access Initiative, New Delhi, India; 3 Northern Arizona University, Center for Health Equity, Flagstaff, Arizona, USA; 4 Teach for America, New York, New York, USA; 5 Alive & Thrive / FHI Solutions LLC, New Delhi, India; 6 RTI International India, New Delhi, India; 7 University of North Carolina at Chapel Hill, Chapel Hill, North Carolina, USA

## Abstract

**Background:**

The Clinton Health Access Initiative implemented a program from 2012-2016 to increase use of oral rehydration salts (ORS) and zinc to treat diarrhea in children under five in three states in India: Gujarat, Madhya Pradesh, and Uttar Pradesh. The program interventions included detailing and development of a rural supply chain to reach private rural health care providers, training of Accredited Social Health Activists (ASHAs), technical support to the state governments, and a mass media campaign targeted at caregivers. In Gujarat and Uttar Pradesh, some of the program activities, such as detailing and ASHA trainings, were targeted to high-burden focal districts, thus providing an opportunity to study their effect compared to statewide activities that covered all districts, such as the mass media campaign. Our study aimed to estimate the effect of activities on ORS and zinc use.

**Methods:**

Household surveys were conducted at two points during the program and in both focal and non-focal districts. We used a difference-in-difference quasi-experimental approach to estimate the effect of the enhanced activities in focal districts and mass media campaign on the odds of a child being treated with ORS and zinc.

**Findings:**

Focal district interventions were associated with a significant increase in the odds of a diarrhea episode receiving ORS in Gujarat and Uttar Pradesh. Living in focal districts increased the odds of receiving ORS in Gujarat and Uttar Pradesh by factors of 3.42 (95% CI = 1.39-8.33) and 2.29 (95% CI = 1.19-4.39), respectively. Focal district interventions were also associated with 15.02 (95% CI = 2.97-75.19) greater odds of receiving both ORS and zinc in Gujarat. In Uttar Pradesh, where the mass media campaign was focused, exposure to the campaign further modified the odds of receiving ORS and combined ORS and zinc by 1.38 (95% CI = 1.04-1.84) and 1.57 (95% CI = 1.01-2.46), respectively.

**Conclusion:**

Comprehensive public and private provider interventions combined with mass media are effective strategies for increasing ORS and zinc use.

Diarrhea is the second largest cause of death in children under five in India, accounting for 9% of all under-five deaths in 2015 [1]. The World Health Organization, Government of India, and Indian Academy of Pediatrics recommend low-osmolarity oral rehydration salts (ORS) and daily zinc supplements for 10-14 days for the treatment of diarrhea in children under five [2]. ORS addresses dehydration, the primary cause of diarrheal deaths, while zinc reduces the reoccurrence and duration of diarrhea. Universal coverage of ORS and zinc is estimated to prevent 93% of diarrheal deaths [3], yet in India, coverage remains low; in 2005-06, coverage of ORS in India was estimated to be 26%, though this varied widely by state, and zinc coverage was 0.3% [4].

Efforts in India to scale-up ORS date back to as early as 1978 when the Government of India started a Diarrheal Disease Control Program, which prioritized treatment with oral rehydration therapy (ORT), environmental sanitation and household hygiene, and child nutrition such as breastfeeding [5]. In 1992, the Ministry of Health and Family Welfare started the Child Survival and Safe Motherhood (CSSM) Programme as part of the Family Welfare Programme. The aim was to have an integrated package of interventions for the betterment of the health status of mothers and children, including treatment of diarrhea with ORS. The Government of India also sponsored special projects under the Maternal and Child Health Programme, including the Oral Rehydration Therapy (ORT) as one of its priority activities for child survival. These and other health programs were integrated into the Reproductive and Child Health Programme in 1996. In 2005, the Government of India launched the National Rural Health Mission (NRHM) to improve availability of and access to quality health care, especially for those residing in rural areas, the poor, women, and children [4,6]. In 2006, the Government of India adopted zinc as an adjunct therapy to ORS.

Non-governmental organizations (NGOs) have also complemented government efforts. Recently, a pilot program called the Diarrhea Alleviation through Zinc Therapy (DAZT) was implemented in 6 districts in Gujarat and 12 districts in Uttar Pradesh from 2011-2014 to improve ORS and zinc uptake [7-9]. The program focused on encouraging use of ORS and zinc among public and private sector providers. In the public sector, the program trained facility–based medical officers and auxiliary nurse midwives (ANMs) and community-level ASHAs and Anganwadi workers (AWWs). In the private sector, the program worked with pharmaceutical partners to detail (ie, one-on-one educational visits) and sell ORS and zinc to formal and informal health care providers. In India, a substantial share of the population seeks care from unlicensed, informal rural health care providers (RHCPs) due to their proximity [10]. An independent evaluation of the program found increased uptake of zinc in both states and ORS in Gujarat. The evaluation cited the lack of community-level demand generation activities as a possible reason for lack of further success in Uttar Pradesh [7].

The Clinton Health Access Initiative (CHAI), with support from the Bill & Melinda Gates Foundation, the IKEA Foundation, and the International Zinc Association implemented a program from 2012 through 2016 in Gujarat, Uttar Pradesh, and Madhya Pradesh to sustainably improve usage of ORS and zinc to treat diarrhea in children under five at state-wide scale. The program expanded upon the DAZT project, which was also funded by the Bill and Melinda Gates Foundation. In particular, the program scaled up activities targeted at public and private providers to 22 districts in Gujarat, all 51 districts in Madhya Pradesh, and 39 districts in Uttar Pradesh. These focal districts included the original 18 districts under the DAZT program. The additional focal districts were selected in collaboration with the state government and other partners to focus on those with poorer performance on health indicators and to coordinate with other donor investments in the state. Data from the District Level Household and Facility Survey 2007-08 (DLHS-3) was used to identify districts with high infant mortality rates, under-five mortality rates, prevalence of diarrhea, low ORS coverage, and low awareness of ORS [11]. **Figure 1** and **Figure 2** present the focal districts covered in Gujarat and Uttar Pradesh, and **Table 1** summarizes the activities by level of geographic coverage.

**Figure 1:**
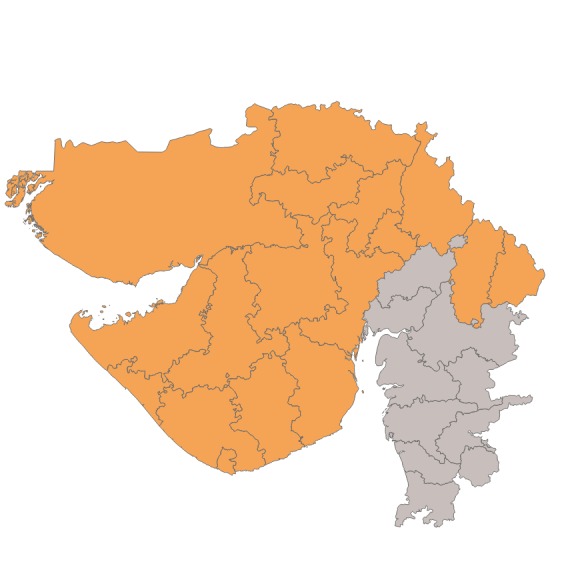
Map of focal districts in Gujarat.

**Figure 2:**
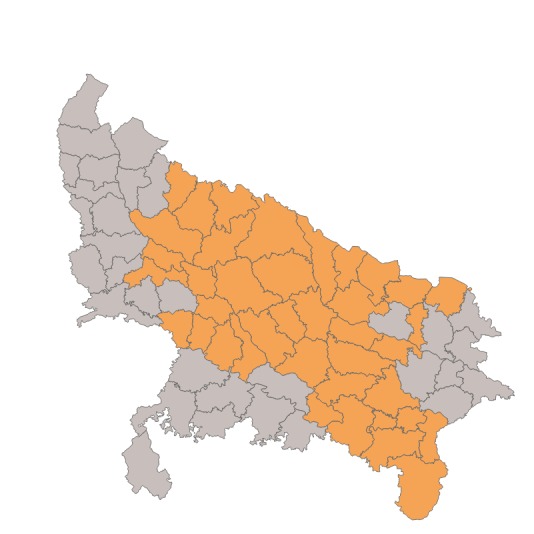
Map of focal districts in Uttar Pradesh.

**Table 1:** Description of program activities in focal areas compared to statewide

	Gujarat	Uttar Pradesh	Madhya Pradesh
Focal districts	- 22 districts (14 districts starting in July 2013 and 8 DAZT districts added in Nov 2013)	- 39 districts in total (26 districts started receiving interventions between April and Dec 2013, 1 district was added in April 2014, and 12 DAZT districts added in June 2015)	- All 51 districts
	- One-on-one detailing with 9600 RHCPs and 2300 chemists	- One-on-one detailing with 61 400 RHCPs and 14 600 chemists	- One-on-one detailing with 49 000 RHCPs and 8000 chemists
		- District-level and Block-level Orientations (DLOs/BLOs) with 14 000 RHCPs	
		- Supportive supervision of 47 000 ASHAs	- Supportive supervision of 55 000 ASHAs
All districts	- Develop diarrhea management module for national and state ASHA training guides		
	- Improved forecasting of ORS and zinc for public facilities and ASHAs		
	- Mass media campaign in Hindi, though with strategic selection of channels targeted to maximize viewership in Madhya Pradesh and Uttar Pradesh		
	- Support national Intensive Diarrhoea Control Fortnite		

Similar to the DAZT program, CHAI’s program partnered with local entrepreneurs to create a sustainable, last-mile supply chain that extended beyond the reach of traditional pharmaceutical distribution networks. The local partners would employ a sales force team to detail and sell ORS and zinc directly to RHCPs in their villages. During the detailing visits, the sales forces used flip charts and videos shown on tablets to provide educational information on the physiology of diarrhea and rationale for using ORS and zinc. At the end of the detailing visit, RHCPs and chemists would have the opportunity to purchase ORS and zinc from the sales representative. The sales teams would also give free posters or notepads to serve as continuous reminders about ORS and zinc. Approximately 120 000 RHCPs and 25 000 chemists were detailed as part of the program. Based on program monitoring data, this is approximately 55% of the total number of RHCPs and chemists in the three states.

Detailing is a common practice in the pharmaceutical industry in developed countries. A systematic review that included 69 studies on detailing found that the visits lead to small, consistent change in health care provider behavior [12]. However, most of the studies included in the systematic review were from high-resource countries. Only three were from low-resource settings. One of the studies in a low-resource setting, a randomized control trial in Nepal, found that detailing improved diarrhea case management; however, this study only involved licensed formal health care providers rather than informal and unlicensed providers like the RHCPs targeted in this program [13]. As discussed above, the DAZT program also included detailing as a primary intervention and the independent evaluation found improved uptake of ORS and zinc. [7,14]. Due to these experiences, detailing continued to be a key feature in the CHAI program.

The program also connected the partners with regional manufacturers of quality assured ORS and zinc. The costs of purchasing ORS and zinc were fully funded by the local partners utilizing the profits from their sales. By reducing the number of hand-offs in the supply chain, this model increased profit margins on sales of ORS and zinc while reducing transport cost due to proximity between the partners and RHCPs. Initially, the program also provided financial support to expand the partners’ sales force and provided free marketing and detailing materials (eg, flip charts, informational leaflets, etc.). The financial support gradually decreased over time, and by mid-2016, the partners were fully self-financing their sales force through profits from sales of ORS, zinc, and a broader basket of goods.

To reinforce the messages from the detailing, the program established and coordinated district-level orientations and block-level orientations (DLOs/BLOs). The DLOs/BLOs were public-private partnerships in which the Governments of Madhya Pradesh and Uttar Pradesh issued letters instructing Chief Medical Officers of the district to conduct meetings with RHCPs to orient them on the use of ORS and zinc as the first-line of treatment for diarrhoea. The orientations between government officials and RHCPs took place at government district and block headquarters. The DLOs/BLOs were implemented between May 2015 and June 2016 and reached approximately 14 000 RHCPs.

In the focal districts of Madhya Pradesh and Uttar Pradesh, the program also organized block-level ASHA trainings that focused on diarrhea case management and quarterly one-on-one mentorship visits to reinforce the trainings. The program helped revise the diarrhea management module of the government’s ASHA training package and trained ASHAs in focal districts on the revised module. Afterwards, the program deployed a field force to meet one-on-one with each ASHA to reinforce the training module using flip charts and videos. In total, the program reached 102 000 ASHAs.

In addition to the various activities implemented in the focal districts, the program also supported several government-led activities to scale-up ORS and zinc beyond the focal districts. The program worked with partners at the WHO, UNICEF, and Micronutrient Initiative to support the Government of India to launch and implement a national Intensified Diarrhoea Control Fortnight (ICDF) program. The core activities under the ICDF program included visits to every household with a child under five by ASHA workers to freely distribute ORS and establishment of ORS-zinc corners in all government facilities. The ICDF program was implemented in 2014-2016 for a 2-week period between July and August, which are months associated with the highest incidence of diarrhea due to the monsoon season. The program provided technical support in providing communication materials and also monitoring of the ICDF activities.

Furthermore, the program implemented a mass media campaign targeted at caregivers. Published studies found that caregiver confidence in ORS as a “medicine” to treat diarrhea was low, and these expectations undermined the confidence of providers to dispense it [15]. The program investigated opportunities to reach caregivers at scale and found through the NFHS-3 and DLHS-3 household surveys that television and radio were common sources of health information for women [4,11]. In the NFHS-3, 42% of rural women reported watching TV at least once a week and 27% reported listening to the radio at least once a week. However, the evidence from literature on the effectiveness of mass media for health behavior change is mixed. Early experiences with using mass media, specifically television, demonstrated success in scaling up ORS in Egypt [16,17]. Analyses from household surveys in India suggested that mass media was significantly associated with knowledge and use of ORS [18,19]. More recently, however, a systematic review of strategies for improving ORS found that while social marketing and mass media had a positive association with ORS use, the effect was not statistically significant, and, a randomized controlled trial of radio messages found no impact on use of ORS [20,21]. Nonetheless, the program implementers believed that combining interventions targeted at health care providers and caregivers would create a “surround sound” effect that would lead to reinforcing behavior change among both groups.

Creative concepts for the mass media campaign were developed based on formative research that uncovered key barriers and insights to drive behavior change in the target population of rural, poor mothers of children under five. The campaign aimed to communicate to caregivers and mothers that using ORS and zinc to treat diarrhea in children leads to a fast and full recovery. The mass media campaign was implemented between August 2015 and October 2016 and included a television advertisement, a radio advertisement, and branded buses traveling through villages. The campaign was aired with high frequency and at peak times of day with the goal of exposing the target audience to the campaign multiple times. The TV advertisement was aired more than 35 000 times on 22 channels. The channels reached all districts in the three states and likely other states in India as well. However, the channels were selected to maximize viewership in Madhya Pradesh and Uttar Pradesh by using data from local marketing agencies on local viewership levels. The language of the campaign messages and channels selected was also Hindi, the primary language in Madhya Pradesh and Uttar Pradesh. The campaign period was chosen to coincide with the rainy season in India, which is also when diarrheal incidence is at its highest throughout the country.

## Study objectives

Our study aimed to estimate the effect of program activities on ORS and combined ORS and zinc use. We first evaluates whether use of ORS and combined ORS and zinc increased during the course of the program. Second, we took advantage of the fact that certain districts in Gujarat and Uttar Pradesh received more intensive support than other districts and evaluate whether there were differences in use of ORS and zinc between focal districts and districts receiving only statewide interventions (“light touch districts”). Lastly, we examined whether the mass media campaign modified the effects of the focal district activities.

## METHODS

### Study design

The primary evaluation activities involved two household survey rounds to measure changes in the study outcomes and potential confounders. The first survey (“early phase”) ran from December 2014 to May 2015 and occurred roughly one year after activities in focal districts began but before the mass media campaign was launched. The second survey (“late phase”) ran from July to October 2016. The study population was children 2-59 months who had diarrhea in the 2 weeks preceding the surveys.

### Sampling design and survey procedures

We used a stratified multi-stage cluster sampling method with probability proportional to household population size (PPS) to select households for the study. The 2011 Indian national census was used as the sampling frame. The sample size for the early phase survey was calculated to detect an approximately ten percentage point change in ORS coverage between survey periods by state. Based on the diarrhea prevalence and ORS coverage from the DLHS-3, we estimated 3300 households per state would be required. In the late phase survey, the sample size was calculated to also test for a ten percentage point difference in ORS and zinc coverage between focal districts and light touch districts. Based on diarrhea prevalence and ORS and zinc coverage estimates found in the early phase survey, we increased the sample size for the late phase survey to 15 960 households for Uttar Pradesh and 7600 households for Gujarat. The detailed breakdown of the sampling plan is provided in **Table 2**.

**Table 2:** Sampling plan

	Early phase (Dec 2014 - May 2015)		Late phase (Jul - Oct 2016)	
**State**	Uttar Pradesh	Gujarat	Uttar Pradesh	Gujarat
**Districts**	20 (7 focal, 13 light touch)	20 (8 focal, 12 light touch)	42 (17 focal, 25 light touch)	20 (14 focal, 6 light touch)
**PSUs**	220	220	798	380
**Households**	3300	3300	15 960	7600

For each data collection period, states were sampled separately as individual strata. Within each stratum, districts were randomly selected using PPS, and within each district, wards (urban) or villages (rural) were selected as the primary sampling unit (PSU) also using PPS. Within the selected PSUs, all households were listed and 20 households in which at least one child under five lived were randomly selected for interview.

The household survey was conducted in person by trained interviewers who received six days of training on topics including survey objectives, interview techniques, and data entry methods. Interviewers also received two days of hands-on practice with the survey in the field before starting data collection. During the household survey interviewers requested to speak with the primary caregiver of children in the household and asked for verbal informed consent prior to beginning the interview. The survey and consent were administered in the local language: Hindi for Uttar Pradesh and Gujarati for Gujarat. The survey was developed in English and translated to Hindi and Gujarati, with the survey design adapted from the Demographic and Health Survey (DHS) and Multiple Indicator Cluster Survey (MICS) model questionnaires [22]. For each child under five in the household, interviewers asked the caregiver whether the child had diarrhea in the two weeks prior to the survey, and if so, additional questions on where care or advice was sought and what treatments were used, including specific questions on whether ORS and zinc were given to the child. Exposure to the media campaign was measured in the household survey by showing the caregiver a short video and audio clip of the campaign ads and asking them whether they had seen or heard the campaign before. Additional information regarding the household, caregiver, and child’s demographic characteristics were also collected.

Independent research agencies – Kantar IMRB for the early phase survey and Mindfield for the late phase – were contracted to carry out the surveys. The research firms were selected using a competitive bidding process. Interviewers were hired directly by the research agencies and were not involved in any aspect of the program implementation.

### Data analysis

#### Variable descriptions

The primary outcomes of the study were whether a child age 2-59 months with diarrhea in the two weeks prior to the survey received ORS or both ORS and zinc. We used binary outcome indicators of whether the child received any ORS or both ORS and zinc to treat diarrhea as reported by the child’s caregiver. There were two binary predictors of interest in this study: first, whether or not the child lived in a focal district; second, whether the child’s caregiver was exposed to the media campaign or not based on the self-reported familiarity with the media campaign.

To build our model we first examined the bivariate associations between ORS use and several potential confounding covariates, including characteristics of the child, household, and caregiver. We did the same bivariate analysis for the outcome of combined ORS and zinc use. Child-level characteristics included the child’s sex, age in years, and whether advice or care was sought for the child’s diarrhea. Age was a categorical variable and included 2-11 months, 1 year old, 2 years old, 3 years old, and 4 years old. For care-seeking, we generated a categorical variable that included ‘did not seek any advice or care outside the home’, ‘sought advice or care in the public sector only’, ‘sought advice or care in the private sector only’, and ‘sought care in both the public and private sector’. Characteristics of the child’s caregiver included categorical variables of sex, age (18-24, 25-29, 30-39, or 40+ years), and whether they had ever received a formal education (yes/no). Household demographics were represented by categorical variables, including whether the household was located in an urban or rural sector and the household wealth status. A household wealth index was constructed using the DHS method utilizing principal components analysis [23]. The final analytic wealth index variable was then categorized into quintiles for each state separately.

### Statistical analysis

First, descriptive statistics were calculated using cross-tabulations of each covariate and outcome by focal and light touch districts. We use χ^2^ tests to check for differences between diarrhea cases in focal districts compared to light touch districts. We then used bivariate logistic regression to test for an association between ORS use or combined ORS and zinc use and each covariate.

The multilevel multivariate logistic regression models were constructed sequentially in three steps. First, we created a multilevel multivariate logistic regression model including all covariates, including potential confounders based on a priori understanding of ORS and zinc use and health behavior in India. All child, caregiver, and household characteristics described above were all included in the model as potential confounders. We then created a second model that included a 2-way interaction of survey period (eg, early phase or late phase) and district intervention category (eg, focal district or light touch district). This 2-way interaction allowed us to solve for the odds of ORS or combined ORS and zinc use in focal districts as compared to light touch districts, accounting for the change between the early and late phase surveys. We did not include a 2-way interaction between media and implementation phase because no caregivers were exposed to the mass media campaign in the early phase, as the mass media campaign did not begin until after the early phase data collection ended. The third model examined the 3-way interaction of survey period, district intervention category, and media exposure. The 3-way interaction allowed us to estimate how the presence of media additionally modified the interaction between focal district interventions and survey period. All models accounted for the complex survey design by adjusting for clustering at the village and household level. We estimated Akaike’s information criterion (AIC) on all three models.

Effects of the interventions were estimated separately from Uttar Pradesh and Gujarat. Madhya Pradesh was excluded from the analysis since focal activities covered the entire state, thus there were no light touch districts for comparison. All analyses were conducted using SAS version 9.4 (SAS Institute, Cary, North Carolina, USA).

### Ethical considerations

The study protocol and tools were reviewed and approved by Advarra in the United States (Pro00017175) and Centre for Media Studies IRB in India (IRB00006230).

## RESULTS

### Sample characteristics

**Figure 3:**
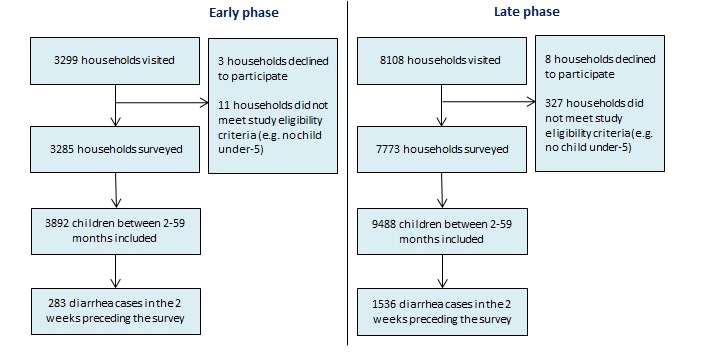
Sampling results for early phase and late phase survey in Gujarat.

**Figure 4:**
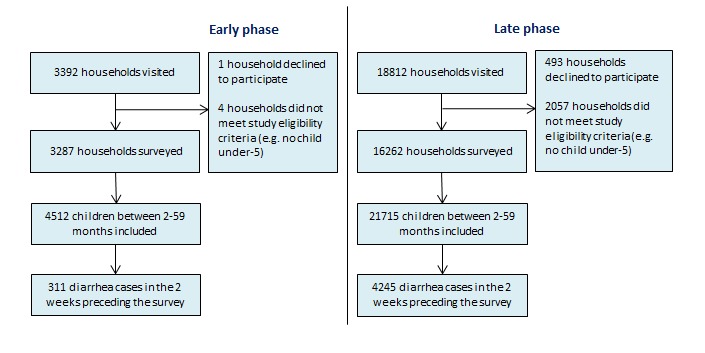
Sampling results for early phase and late phase survey in Uttar Pradesh.

**Figure 3** and **Figure 4** present our sampling results. In total, we visited 33 611 households. 505 households refused to participate in the study while 2399 did not meet the study inclusion criteria resulting in 30 607 completed household surveys. We found 39 607 children 2-59 months living within those households, of which 6375 had diarrhea in the 2 weeks preceding the survey. Diarrhea prevalence was lower in the early phase survey than in the late phase survey in both states. In Gujarat, we found a diarrhea prevalence of 7% (95% CI = 6-8%) in the early phase survey and 16% (95% CI = 15-17%) in the late phase survey. In Uttar Pradesh, the 2-week prevalence of diarrhea was found to be 7% (95% CI = 6-8%) and 20% (95% CI = 19-20%) in the early phase and late phase survey, respectively. The difference in diarrhea prevalence found between the two surveys can be explained by the difference in when the surveys were conducted. The early phase survey was conducted during the dry months while the late phase survey was conducted during the monsoon and rainy months which are associated with higher diarrhea incidence.

**Table 3** presents the distribution of diarrhea episode characteristics by district intervention category in the early phase survey. In Gujarat, treatment with ORS and treatment with combined ORS and zinc were not significantly different between diarrhea cases living in focal districts compared to light touch districts. Most other covariates were also not significantly different. However, a greater proportion of diarrhea cases living in light touch districts were from rural villages compared to focal districts (*P* = 0.05). Diarrhea cases from light touch districts were also poorer than diarrhea cases in focal districts (*P* = 0.01).

**Table 3:** Distribution of sample characteristics by detailing status in early phase survey

	Gujarat (n %)			Uttar Pradesh (n, %)		
**Variables**	**Not detailed (N = 142)**	**Detailed (N = 141)**	***P*-value**	**Not detailed (N = 195)**	**Detailed (N = 116)**	***P*-value**
**Outcome**						
ORS use	84 (59.15)	83 (58.87)	0.96	65 (33.33)	29 (25.00)	0.12
ORS and zinc use	14 (9.86)	20 (14.18)	0.27	10 (5.13)	6 (5.17)	0.99
**Child characteristics**						
Female	61 (42.96)	59 (41.84)	0.85	90 (46.15)	55 (47.41)	0.84
Age (in completed years):						
0	36 (25.35)	36 (25.53)	0.99	65 (33.33)	29 (25.00)	0.11
1	47 (33.10)	48 (34.04)		55 (28.21)	42 (36.21)	
2	27 (19.01)	28 (19.86)		36 (18.46)	29 (25.00)	
3	17 (11.97)	16 (11.35)		25 (12.82)	8 (6.90)	
4	15 (10.56)	13 (9.22)		14 (7.18)	8 (6.90)	
Source of care outside the home:						
- Did not seek care outside the home	67 (47.18)	72 (51.06)	0.88	34 (17.44)	19 (16.38)	0.86
- Sought public care only	13 (9.15)	12 (8.51)		13 (6.67)	5 (4.31)	
- Sought private care only	54 (38.03)	52 (36.88)		130 (66.67)	82 (70.69)	
- Sought both public and private care	8 (5.63)	5 (3.55)		18 (9.23)	10 (8.62)	
**Caregiver characteristics**						
Female	134 (94.37)	128 (90.78)	0.38	189 (96.92)	109 (93.97)	0.22
Age:						
18-24	52 (36.62)	50 (35.46)	0.89	57 (29.23)	22 (18.97)	0.14
25-29	62 (43.66)	59 (41.84)		76 (38.97)	44 (37.93)	
30-39	20 (14.08)	25 (17.73)		46 (23.59)	35 (30.17)	
40+	8 (5.63)	7 (4.96)		16 (8.21)	15 (12.93)	
Education:						
- Never been to school	49 (34.51)	32 (22.70)	0.06	89 (45.64)	58 (50.00)	0.51
-Ever been to school	93 (65.49)	109 (77.30)		106 (54.36)	58 (50.00)	
Exposed to mass media campaign	0 (0.00)	0 (0.00)	-	0 (0.00)	0 (0.00)	-
**Household characteristics**						
Rural	104 (73.24)	78 (55.32)	0.05	153 (78.46)	102 (87.93)	0.18
Wealth quintile:						
- Highest	19 (13.38)	27 (19.15)	0.01	32 (16.41)	14 (12.07)	0.05
- Fourth	21 (14.79)	36 (25.53)		47 (24.10)	20 (17.24)	
- Middle	30 (21.13)	30 (21.28)		35 (17.95)	20 (17.24)	
- Second	33 (23.24)	34 (24.11)		51 (26.15)	25 (21.55)	
- Lowest	39 (27.46)	14 (9.93)		30 (15.38)	37 (31.90)	

In Uttar Pradesh, there was no difference between focal and light touch districts in the proportion of diarrhea cases receiving either ORS (*P* = 0.12) or ORS and zinc (*P* = 0.99). Like Gujarat, most covariates were not significantly different between focal and light touch districts. Among our selected covariates, the distribution of diarrhea cases among wealth quintiles was significantly different between focal and light touch districts with more diarrhea cases in focal districts living in poorer households (*P* = 0.05).

In **Table 4** we also present the distribution of a child receiving either ORS or combined ORS and zinc by the selected characteristics and crude, unadjusted measures of association. In unadjusted logistic regressions of the primary predictors for Gujarat, living in a focal district (cOR = 2.26; 95% CI = 1.55-3.29) and exposure to mass media campaign (cOR = 1.76; 95% CI = 1.35-2.29) were all significantly associated with higher odds of the child receiving ORS. The late phase survey period was associated with 0.88 (95% CI = 0.58-1.33) lower odds of receiving ORS, though this was not statistically significant (*P* = 0.54). With respect to combined ORS and zinc use in Gujarat, the late phase survey period (cOR = 6.77; 95% CI = 3.23-14.17), living in a focal district (cOR = 14.29; 95% CI = 7.08-28.86), and being exposed to the mass media campaign (cOR1.98; 95% CI = 1.36-2.86) were all associated with higher odds of a child receiving combined ORS and zinc.

**Table 4:** Distribution of sample characteristics and treatment outcome by covariates

GUJARAT (N = 1819)								
	**Outcome: ORS use**			**Outcome: ORS and zinc use**				
**Variables**	**%**	**cOR (95% CI)**	***P*-value**	**%**	**cOR (95% CI)**		***P*-value**	**n**
**Primary predictors**								
Survey round:								
- Early phase	59.01	Ref		12.01	Ref			283
- Late phase	53.65	0.88 (0.58-1.33)	0.54	27.67	6.77 (3.23-14.17)		<0.01	1536
District intervention category:								
- Light touch district	43.42	Ref		6.79	Ref			486
- Focal district	58.51	2.26 (1.55-3.29)	<0.01	31.96	14.29 (7.08-28.86)		<0.01	1333
Exposure to mass media campaign:								
- No	51.96	Ref		22.85	Ref			1,247
- Yes	59.97	1.76 (1.35-2.29)	<0.01	30.42	1.98 (1.36-2.86)		<0.01	572
**Child characteristics**								
Gender:								
- Male	52.84	Ref		24.78	Ref			1,005
- Female	56.51	1.22 (0.97-1.53)	0.09	25.80	1.13 (0.83-1.54)		0.43	814
Age (in completed years):								
- 0	49.26	Ref		20.05	Ref			404
- 1	51.49	1.22 (0.89-1.68)	0.21	24.55	2.09 (1.31-3.32)		<0.01	505
- 2	58.97	1.53 (1.08-2.18)	0.02	28.21	1.78 (1.08-2.93)		0.02	351
- 3	57.39	1.55 (1.07-2.25)	0.02	22.89	1.38 (0.81-2.34)		0.23	284
- 4	58.91	1.53 (1.05-2.24)	0.03	32.73	2.16 (1.29-3.62)		<0.01	275
Source of care outside the home:								
- Did not seek care outside the home	38.63	Ref		8.10	Ref			321
-Sought public care only	71.34	6.61 (4.23-10.32)	<0.01	38.22	11.26 (5.77-21.99)		<0.01	314
- Sought private care only	54.66	2.61 (1.85-3.67)	<0.01	26.85	5.14 (2.85-9.27)		<0.01	1,147
- Sought both public and private care	43.24	1.81 (0.77-4.27)	0.17	13.51	3.43 (0.86-13.71)		0.08	37
**Caregiver characteristics**								
Gender:								
- Male	55.56	Ref		15.56	Ref			45
- Female	54.45	0.91 (0.43-1.94)	0.81	25.48	2.05 (0.66-6.37)		0.22	1,774
Age:								
- 18-24	49.81	Ref		20.15	Ref			536
- 25-29	59.22	1.34 (1.02-1.76)	0.04	28.61	1.41 (0.96-2.07)		0.08	748
- 30-39	54.99	1.09 (0.79-1.51)	0.58	29.41	1.65 (1.05-2.58)		0.03	391
- 40+	45.83	1.07 (0.68-1.68)	0.78	15.28	0.78 (0.38-1.59)		0.48	144
Education:								
- Never been to school	47.26	Ref		19.09	Ref			529
- Ever been to school	57.44	1.51 (1.17-1.96)	<0.01	27.75	2.12 (1.44-3.12)		<0.01	1,290
**Household characteristics**								
Rural/Urban:								
- Rural	53.08	Ref		26.25	Ref			1,219
- Urban	57.33	1.21 (0.85-1.74)	0.29	23.17	0.92 (0.51-1.69)		0.79	600
Wealth quintile:								
- Highest	65.22	Ref		32.25	Ref			276
- Fourth	56.19	0.67 (0.45-1.01)	0.06	25.75	0.63 (0.35-1.09)		0.09	299
- Middle	54.24	0.66 (0.45-0.99)	0.04	24.94	0.59 (0.35-1.00)		0.05	413
- Second	49.27	0.5 (0.33-0.74)	<0.01	23.90	0.44 (0.25-0.77)		<0.01	410
- Lowest	51.54	0.6 (0.40-0.91)	0.01	21.85	0.43 (0.24-0.77)		<0.01	421
**UTTAR PRADESH (N = 4556)**								
	**Outcome: ORS use**			**Outcome: ORS and zinc use**				
**Variables**	**%**	**cOR (95% CI)**	***P*-value**	**%**		**cOR (95% CI)**	***P*-value**	**n**
**Primary predictors**								
Survey round:								
- Early phase	30.23	Ref		5.14		Ref		311
-Late phase	44.33	1.99 (1.47-2.69)	<0.01	12.01		2.57 (1.47-4.49)	<0.01	4,245
District intervention category:								
- Light touch district	39.36	Ref		9.97		Ref		2,467
- Focal district	48.11	1.51 (1.28-1.79)	<0.01	13.40		1.49 (1.16-1.91)	<0.01	2,089
Exposure to mass media campaign:								
- No	34.94	Ref		7.45		Ref		2,281
- Yes	51.82	2.01 (1.75-2.29)	<0.01	15.65		2.39 (1.93-2.95)	<0.01	2,275
**Child characteristics**								
Gender:								
- Male	44.49	Ref		11.72		Ref		2,414
- Female	42.11	0.91 (0.80-1.03)	0.13	11.34		0.98 (0.80-1.19)	0.82	2,142
Age (in completed years):								
- 0	40.00	Ref		11.56		Ref		1,090
- 1	43.98	1.25 (1.04-1.50)	0.01	12.92		1.2 (0.92-1.58)	0.18	1,246
- 2	48.01	1.48 (1.21-1.79)	<0.01	10.98		0.97 (0.72-1.31)	0.84	929
- 3	41.49	1.12 (0.90-1.39)	0.32	11.04		0.97 (0.70-1.34)	0.83	670
- 4	43.16	1.25 (1.00-1.56)	0.05	10.14		0.9 (0.64-1.27)	0.56	621
Source of care outside the home:								
- Did not seek care outside the home	25.65	Ref		4.66		Ref		386
- Sought public care only	74.80	9.46 (6.35-14.09)	<0.01	35.20		11.88 (6.68-21.12)	<0.01	250
- Sought private care only	44.00	2.55 (1.96-3.31)	<0.01	10.80		2.57 (1.56-4.24)	<0.01	3,732
- Sought both public and private care	25.53	1.01 (0.66-1.56)	0.96	9.04		2.14 (1.04-4.39)	0.04	188
**Caregiver characteristics**								
Gender:								
- Male	40.82	Ref		4.08		Ref		49
- Female	43.40	1.07 (0.57-2.02)	0.84	11.63		2.97 (0.69-12.85)	0.14	4,507
Age:								
- 18-24	45.36	Ref		11.43		Ref		1,164
- 25-29	43.92	0.96 (0.81-1.12)	0.57	12.11		1.07 (0.84-1.37)	0.61	1,940
- 30-39	41.80	0.92 (0.77-1.10)	0.35	10.67		0.96 (0.73-1.26)	0.75	1,275
- 40+	35.59	0.65 (0.45-0.93)	0.02	12.43		1.09 (0.64-1.84)	0.76	177
Education:								
- Never been to school	36.98	Ref		9.30		Ref		2,001
- Ever been to school	48.38	1.61 (1.40-1.84)	<0.01	13.31		1.54 (1.25-1.89)	<0.01	2,555
**Household characteristics**								
Rural/Urban:								
- Rural	42.08	Ref		11.73		Ref		3,897
- Urban	50.99	1.44 (1.13-1.84)	<0.01	10.47		0.88 (0.61-1.28)	0.51	659
Wealth quintile:								
- Highest	57.09	Ref		14.55		Ref		550
- Fourth	49.62	0.72 (0.56-0.92)	<0.01	14.70		0.97 (0.69-1.36)	0.85	796
- Middle	44.98	0.6 (0.47-0.77)	<0.01	12.50		0.75 (0.53-1.06)	0.11	976
- Second	39.50	0.47 (0.37-0.60)	<0.01	9.56		0.57 (0.40-0.81)	<0.01	1,109
- Lowest	34.67	0.38 (0.29-0.48)	<0.01	8.89		0.52 (0.36-0.74)	<0.01	1,125

ORS – oral rehydration salts

In Uttar Pradesh, we found that the late phase survey period (cOR 1.99; 95% CI = 1.51-2.69), living in a focal district (cOR = 1.51; 94% CI = 1.28-1.79), and exposure to the mass media campaign (cOR = 2.01; 95% CI = 1.75, 2.29) were all associated with higher odds of a child receiving ORS. For combined ORS and zinc in Uttar Pradesh, we similarly found higher odds associated with the late phase survey period (cOR = 2.57; 95% CI = 1.47-4.49), living in a focal district (cOR = 1.49; 95% CI = 1.16-1.91), and exposure to the mass media campaign (cOR 2.39; 95% CI = 1.93-2.95).

### Multilevel multiple logistic regression

In **Table 5**, we present our multilevel multivariate regression results. For each state and outcome, we created three models: the first without an interaction term, the second with a 2-way interaction term between implementation phase and detailing status, and the third with a 3-way interaction term between implementation phase, detailing status, and media exposure. Adjusting for changes over time and other potential confounders, we find focal district interventions significantly increased the odds of a diarrhea episode receiving ORS in Gujarat (*P* < 0.01) and Uttar Pradesh (*P* = 0.01) between the survey periods. Focal district interventions in Gujarat increased the odds of receiving ORS by a factor of 3.42 (95% CI = 1.39-8.33) between the early phase survey and late phase survey. In Uttar Pradesh, living in focal districts elevated the odds of receiving ORS by a factor of 2.29 (95% CI = 1.19-4.39). For the odds of receiving both ORS and zinc, we found a statistically significant association with focal district interventions in Gujarat (*P* < 0.01), after adjusting for changes over time and other confounders. In Gujarat, focal district interventions increased the odds of receiving combined ORS and zinc by a factor of 15.02 (95% CI = 2.97-75.19) between survey periods. Finally, in Uttar Pradesh, the mass media campaign further modified the effect of focal district interventions by a factor of 1.38 (95% CI = 1.04-1.84) for receiving ORS and 1.57 (95% CI = 1.01-2.46) for combined ORS and zinc treatment.

**Table 5:** Multilevel multivariate logistic regression analyses of the association between interventions and receipt of ORS and zinc

GUJARAT								
	**Outcome: ORS use**				**Outcome: ORS and zinc use**			
	Model 1* aOR (95% CI)	**Model 2† aOR (95% CI)**	**Model 3‡ aOR (95% CI)**	***P*-value for Model 3**	Model 1* aOR (95% CI)	**Model 2† aOR (95% CI)**	**Model 3‡ aOR (95% CI)**	***P*-value for Model 3**
**Main effects**								
Survey round								
Early phase	Ref				Ref			
Late phase	0.33	0.31	0.17	<0.01	1.86	1.26	0.31	0.08
	(0.21, 0.53)	(0.19, 0.49)	(0.08, 0.34)		(0.85, 4.08)	(0.56, 2.83)	(0.08, 1.15)	
								
District intervention category								
Light touch district	Ref				Ref			
Focal district	2.48	1.88	0.93	0.85	11.06	6.86	1.89	0.31
	(1.65, 3.72)	(1.21, 2.90)	(0.45, 1.93)		(5.28, 23.19)	(3.15, 14.95)	(0.55, 6.49)	
								
Mass media exposure								
Not exposed to mass media campaign	Ref				Ref			
Exposed to mass media campaign	1.75	1.73	1.19	0.58	1.41	1.39	1.92	0.26
	(1.32, 2.32)	(1.31, 2.29)	(0.67, 2.07)		(0.96, 2.06)	(0.95, 2.04)	(0.62, 5.93)	
								
**Interactions**								
Survey round * Focal district	N/A	4.01	3.42	<0.01	N/A	12.93	15.02	<0.01
		(1.68, 9.68)	(1.39, 8.33)			(2.80, 60.34)	(2.97, 75.19)	
								
Survey round * Focal district * Mass media	N/A	N/A	1.66	0.12	N/A	N/A	0.69	0.56
			(0.86, 3.16)				(0.21, 2.32)	
								
**Model fit - AIC (lower is better)**	2226.63	2218.78	2218.48		1539.06	1530.35	1532.00	
								
**UTTAR PRADESH**								
	**Outcome: ORS use**				**Outcome: ORS and zinc use**			
	Model 1*	**Model 2†**	**Model 3‡**	**p-value**	Model 1*	**Model 2†**	**Model 3‡**	**p-value**
	**aOR**	**aOR**	**aOR**	**for**	**aOR**	**aOR**	**aOR**	**for**
	**(95% CI)**	**(95% CI)**	**(95% CI)**	**Model 3**	**(95% CI)**	**(95% CI)**	**(95% CI)**	**Model 3**
**Main effects**								
Survey round								
Early phase	Ref				Ref			
Late phase	1.36	1.55	1.02	0.91	1.59	1.64	1.61	0.20
	(1.00-1.86)	(1.12-2.17)	(0.70-1.51)		(0.89-2.84)	(0.90-2.98)	(0.77-3.38)	
								
District intervention category								
Light touch district	Ref				Ref			
Focal district	1.65	1.08	0.66	0.17	1.44	1.24	1.72	0.92
	(1.40-1.96)	(0.79-1.49)	(0.36-1.20)		(1.13-1.84)	(0.70-2.22)	(0.34-3.25)	
								
Mass media exposure								
Not exposed to mass media campaign	Ref				Ref			
Exposed to mass media campaign	1.56	1.56	1.34	<0.01	1.94	1.95	1.54	<0.01
	(1.34-1.82)	(1.34-1.82)	(1.09-1.63)		(1.53-2.47)	(1.53-2.47)	(1.12-2.11)	
								
**Interactions**								
Survey round * Focal district	N/A	2.74	2.29	0.01	N/A	1.37	1.02	0.97
		(1.45-5.16)	(1.19-4.39)			(0.44-4.35)	(0.31-3.35)	
								
Survey round * Focal district * Mass media	N/A	N/A	1.38	0.02	N/A	N/A	1.57	0.04
			(1.04-1.84)				(1.01-2.46)	
								
**Model fit - AIC (lower is better)**	5778.20	5770.18	5767.13		3037.65	3039.34	3037.20	

ORS – oral rehydration salts, aOR – adjusted odds ratio, AIC – Akaike’s information criterion

*Model 1 is a multivariate logistic regression model including all covariates.

†Model 2 is a multivariate logistic regression model including all covariates and a 2-way interaction between survey round and focal district interventions.

‡Model 3 is a multivariate logistic regression model including all covariates, a 2-way interaction between survey round and detailing, and a 3-way interaction between survey round, focal district interventions, and mass media exposure.

§All models are adjusted for child gender, child age, source of care, respondent education level, urban/rural, and household wealth status.

## DISCUSSION

Our results demonstrate that focal district interventions were significantly associated with increased use of ORS to treat diarrhea in children under five. In Uttar Pradesh, the results also show that targeted mass media had an amplifying effect, further increasing the odds of ORS and zinc treatment. The results from this study support the program approach of addressing supply and demand via engagement of providers and caregivers to achieve the greatest results. More specifically, it demonstrates that implementing layered interventions (here, mass media targeted at caregivers on top of high-frequency training interventions targeted at health care providers) increases the positive potential effects more than single interventions in isolation (here, trainings and detailing without mass media).

In Uttar Pradesh, we did not observe a significant effect of program interventions on combined ORS and zinc treatment (*P* = 0.97). This is likely due to underreporting of treatment with zinc in Uttar Pradesh. In the household survey, we found that a large proportion of caregivers reported treating their child’s diarrhea episode with “unknown tablets”, ie, tablets that had been removed from their original packaging and the name of the medicine was unknown to the respondent. This is a common practice in India as patients may only be able to afford a few tablets at a time. During the household survey, we used picture cards to help respondents identify the medicines used to treat their child’s diarrhea and also asked to see the packaging, if there was any. Despite these efforts, 47% of all diarrhea cases in Uttar Pradesh reported receiving “unknown tablets” compared to only 10% in Gujarat. As part of a separate study conducted during the late phase of the program, we determined that approximately 25% of tablets dispensed by RHCPs and chemists for a diarrhea episode in Uttar Pradesh were likely zinc though only 12% of diarrhea episodes in the household survey reported receiving zinc in the household survey.

Adjusting for other covariates, the mass media campaign was found to be significantly associated with increased odds of treatment in Uttar Pradesh, where the campaign was focused. As mentioned above, the mass media campaign was designed specifically for Uttar Pradesh due to its high burden of diarrhea and low coverage of ORS and zinc. This included selection of channels for airing the campaign and use of Hindi for the message’s language. The program purchased time on channels that reached the most viewers in Uttar Pradesh. These channels are also available in Gujarat and so the campaign messages were also seen and heard by caregivers in Gujarat, but only 31% of Gujarat caregivers whose child had an episode of diarrhea reported hearing or seeing the mass media campaign. In Uttar Pradesh, 50% of caregivers whose child had diarrhea reported hearing or seeing the mass media campaign. The different results found in Uttar Pradesh compared to Gujarat suggest that for a mass media campaign to be effective, the mass media campaign should be designed for the local context and requires consistent messaging at a high frequency.

Limitations of the study design include non-randomized assignment of focal districts to receive the program intensive interventions. As mentioned previously, the program collaborated with the government and other partners to purposely select poorer performing districts to receive the focal interventions. Thus, the study population in focal districts may not be comparable to those in light touch districts. We did find initial differences between diarrhea episodes living in focal and light touch districts. Though the multiple logistic regressions include several measured confounders to account for differences that might induce bias to our models, we cannot conclusively rule out the role of unmeasured confounding on the differences between focal and light touch districts.

Our evaluation approach used a difference-in-difference quasi-experimental design to estimate the effect of focal district interventions. Our multivariate logistic regression model included an interaction term between survey round and focal district to estimate the difference in the odds of receiving ORS and zinc between focal and light touch districts while adjusting for non-intervention related changes in the outcome over time. One important pre-requisite for conducting a difference-in-difference estimate is that focal and light touch districts have parallel trends over time in the outcome prior to the intervention. We have no reason to believe that trends in ORS and zinc use in the detailed districts and non-detailed districts would be significantly different prior to our program. Prior household surveys such as the NFHS-2, NFHS-3, DLHS-2, and DLHS-3 showed ORS use had not changed in Uttar Pradesh or Gujarat between 1998 and 2007 [4,11,24,25]. The surveys estimated ORS coverage in Gujrat was 29% in 1998, 24% in 2002, 26% in 2005, and 37% in 2007. Though there appears to be an increase in ORS coverage in 2007, secondary analysis shows the estimate is not statistically different from any of the estimates from prior years. In Uttar Pradesh, ORS coverage similarly had no change: 16% in 1998, 15% in 2002, 13% in 2005, and 17% in 2007. Zinc was only recently included as part of the recommended treatment in 2006 so it was unlikely to have been different in focal and light touch districts. However, given that we only collected data at 2 time points, we are unable to confirm that trends in ORS and zinc use between focal and light touch districts prior to the program were identical.

Additionally, the focal district interventions had been rolled out approximately a year prior to the early phase survey and could have affected ORS and zinc use prior to this survey. We assume that the program interventions would have increased ORS and zinc use from the program’s start, which would imply that the results from this study may actually underestimate the program’s effect as presented here.

The findings also relied on self-reported information by caregivers of children. We tried to limit the potential for bias induced by poor caregiver recall by asking about diarrhea episodes occurring two weeks prior to the survey. This method is widely accepted and used in other population-based household surveys such as the DHS and MICS. In addition, data collectors presented caregivers with flipcharts of locally branded treatments, including ORS, zinc, antibiotics, anti-diarrheals, and home remedies, to aid in recall of the treatment used for the diarrhea episode.

We also note that the use of the complex sampling procedure calls for additional weighting of the data. However, incorporating complex survey weights into multilevel random effects models is an emerging, computationally intensive statistical technique only recently available in statistical software packages. As such, we did not include sampling weights in our multilevel analyses.

This study focused only on two states in India. Care seeking for diarrhea in children under five in both Uttar Pradesh and Gujarat was relatively high with about 90% of all diarrhea cases seeking care by the late phase survey, and the majority of caregivers seeking care from the private sector. This unique context may explain why this study was able to demonstrate that such interventions could successfully translate to treatment outcomes even on the individual level. Our results may not be generalizable to other Indian states or other countries where population characteristics, media penetration, and care seeking behaviors differ significantly. Furthermore, the specific barriers to proper treatment of childhood diarrhea are likely to vary across contexts and should be considered when designing and implementing programs.

Lastly, we acknowledge that the study involved members of CHAI which may introduce an inherent bias. Even though several authors were part of the implementing team, we’re confident that our study design was rigorous and that an external evaluator would find the same results. We attempted to mitigate the bias by hiring an external research agency to oversee the data collection. Additionally, we have compared our survey results with independent external data sources. The NFHS-4 was conducted in between the early phase and late phase surveys [26]. The coverage estimates from the NFHS-4 for the states of Gujarat and Uttar Pradesh are close to the results found in our surveys. In Gujarat, the NFHS-4 found state ORS and combined ORS and zinc coverage estimates of 46% and 17%, respectively. As shown in **Table 4**, we found ORS coverage estimates of 59% in the early phase survey, which was conducted approximately 1 year prior to the NFHS-4, and 54% in the late phase survey, several months after the NFHS-4. Combined ORS and zinc coverages estimates in Gujarat were 12% in the early phase survey and 28% in the late phase survey. In Uttar Pradesh, the NFHS-4 found ORS and combined ORS and zinc coverage estimates of 38% and 13%, respectively. As shown in **Table 4**, we found ORS coverage estimates of 30% in the early phase survey and 44% in the late phase survey. For combined ORS and zinc coverage, we found coverage estimates of 5% in the early phase survey and 12% in the late phase survey.

The study limitations notwithstanding, we found the results of the study consistent with monitoring data from the program. While not presented here, the program monitoring data showed increasing availability and dispensing of ORS and zinc amongst the RHCPs and ASHAs. In addition to improving provider knowledge and prescription practices of ORS and zinc, the focal district interventions also aimed to improve the availability of these commodities at the community level and to strengthen the last-mile supply chain. In the private sector, as detailers visited the RHCPs, they were given the opportunity to purchase ORS and zinc during each detailing visit. Thus, we found availability of both commodities increase over the duration of the program. Perhaps more importantly, the monitoring data showed that by the late phase of the program, a greater percentage of RHCPs and chemists in focal districts (60% in Uttar Pradesh and 73% in Gujarat) were stocking ORS than those in light touch districts (42% in Uttar Pradesh and 52% in Gujarat). The same trend in stocking behavior was observed when looking at both ORS and zinc: more RHCPs in focal districts had both commodities in stock (53% in Uttar Pradesh and 66% in Gujarat) than in light touch districts (23% in Uttar Pradesh and 50% in Gujarat). These trends suggest that focal district interventions likely also contributed to an increase in availability of the commodities which in turn may have influenced ORS and zinc use in these districts.

The results from this study add to the body of evidence on effective program interventions and designs for improving use of ORS and zinc [6,7,9,13,27-32]. Most prior program evaluation studies were either smaller pilots aimed to test specific interventions or did not include comparison areas. This study builds on previous ORS and zinc program evaluation methodologies by evaluating differing levels of intervention intensities. Our study contributes to the existing literature by providing evidence that the focal district interventions were associated with population-level improvements in treatment use and can have a greater impact when combined with a demand-side intervention like mass media.

More research is needed on the impact of detailing, provider trainings, mass media, and multi-layered programs in other low resource settings as well as on other outcome measures to further understand how detailing and mass media can best be used to drive usage of recommended treatment and improve health outcomes. Areas of further research may also include examining the frequency and duration of detailing visits to assess the extent to which each additional detailing visit affects the desired behavior change and if there is a threshold for diminishing returns. Future studies could use a factorial randomized control or step-wedge design to allow for rigorous study of each intervention in true isolation as well as the combined interventions and be able to directly compare results across these treatment arms.

## CONCLUSION

Diarrhea remains a major cause of childhood morbidity and mortality in India. Comprehensive program activities, particularly intensive public and private provider detailing and supportive supervision, can be effective interventions to increase the use of ORS and zinc, and the addition of a media campaign on top of these interventions can have an additional amplifying effect on increased ORS and zinc treatment.
